# Selective Neuroendoscopic Resection of the Choroid Plexus as an Alternative Technique for Optimizing the Standard Endoscopic Approach to Hydrocephalus

**DOI:** 10.7759/cureus.11618

**Published:** 2020-11-22

**Authors:** Leopoldo Mandic Ferreira Furtado, José Aloysio Da Costa Val Filho, Camila Moura de Sousa, François Dantas, Julia Braga Holliday

**Affiliations:** 1 Pediatric Neurosurgery, Hospital Vila da Serra, Nova Lima, BRA; 2 Neurosurgery, Hospital Vila da Serra, Nova Lima, BRA; 3 Neurosurgery, Federal University of Vales do Jequitinhonha e Mucuri, Diamantina, BRA; 4 Neurosurgery, Biocor Instituto, Belo Horizonte, BRA; 5 Neurosurgery, Hospital Vila da Serra, Belo Horizonte, BRA; 6 Pediatric Neurosurgery, José Rosário Vellano University, Hospital Vila da Serra, Belo Horizonte, BRA

**Keywords:** endoscopic third ventriculostomy, choroid plexus coagulation, plexectomy, hydrocephalus

## Abstract

In the past four decades, enormous advances have been made in the neuroendoscopic techniques, along with improvement of illumination, and the development of effective instruments. As a result, endoscopic third ventriculostomy (ETV) and choroid plexus cauterization (CPC) have become consolidated techniques for the treatment of hydrocephalus. In particular, endoscopic cauterization of the choroid plexus has increased the effectiveness of hydrocephalus treatment in combination with ETV. In the past decade, the use of flexible endoscopes has enabled surgeons to resect even the temporal segment of the choroid plexus at the lateral ventricles, which has increased the success of treatment. In this technical note, we describe CPC with the use of a rigid endoscope, which we used to selectively disconnect the glomus of the choroid plexus, in addition to choroid plexus coagulation, as an alternative way to facilitate ETV. This new procedure optimized the visualization of the choroid plexus and the temporal horn and prevented additional difficulties in coagulation of this mobile region of the choroid plexus in selected patients. To achieve the best outcome, avoid bleeding, and optimize the standard technique, it was important to recognize both the classical anatomic structure of the choroid plexus and some variations, and previous expertise in ETV and CPC were necessary. We demonstrate that resection of the glomus of the choroid plexus in selected patients is safe and feasible.

## Introduction

Since Victor Lespinasse, using a cystoscope, performed the first fulguration of the choroid plexus in two young children at the beginning of the 20th century, ventricular neuroendoscopy has been used in the treatment of hydrocephalus [1]. In 1922, Walter Dandy used a cystoscope positioned at the parietal bone to performed the first resection of the choroid plexus, disconnecting it from the surface of ventricles; however, the results were unsatisfactory, inasmuch as ventricular collapse and excessive bleeding ensued, and three of the four patients died one month after the operation [2]. The history of neuroendoscopy was subsequently characterized by several innovations such as third ventriculostomy, first performed by William J. Mixter in 1923; improvements in neuroendoscopic design by John E. Scarff and Tracy J. Putnam; and, in the mid-1900s, the creation of the red rod lens by Harold H. Hopkins, which greatly improved the visualization of the ventricular anatomy [1]. Because of numerous poor outcomes of plexectomy and choroid plexus cauterization (CPC), it was not until the end of the 20th century, after more improvements and refinements in the armamentarium and in neuroendoscopic technique, that endoscopic third ventriculostomy (ETV) could be performed safely as treatment of hydrocephalus [1,3].

Endoscopic CPC came back into use after the cornerstone research of Warf [4], who reported the combination of ETV and CPC with an excellent success rate in the treatment of hydrocephalus. He also reported some advantages and the effectiveness of using a flexible endoscope instead a rigid endoscope during CPC in the temporal horn.

Nevertheless, during CPC, coagulation of the glomus of the choroid plexus (GCP) is difficult with both rigid and flexible endoscopes, especially in cases of myelomeningocele with associated colpocephaly and a mobile GCP; such anatomic disorders could influence in the effectiveness of this procedure.

The purpose of this technical note is to describe our adaptation of endoscopic technique, in which we used the classical concepts of choroid plexus extirpation initially advocated by Dandy and improved upon by others, in combination with CPC. We performed selective endoscopic extirpation of the GCP in addition to classical CPC and ETV in selected patients. We also describe an anatomic classification of GCP that may facilitate the selection of patients to undergo our procedure.

## Technical report

Patient selection

This technique can be applied only in patients with a mobile and elongated GCP. In our practice, we have identified five fundamental types of choroid plexuses on endoscopic view, which were corroborated by magnetic resonance imaging (MRI) (Table 1).

**Table 1:** Anatomic variations of glomus of choroid plexus on the endoscopic view

Glomus of choroid plexus	Type 1	Type 2	Type 3	Type 4	Type 5
Mobilization	Mobile	Mobile	Mobile	Unmobile	Absent
Elongation	Minimal	Moderate	Extreme	Absent	Absent
Endoscopic resection is suitable	No	Yes	Yes	No	No

**Figure 1:**
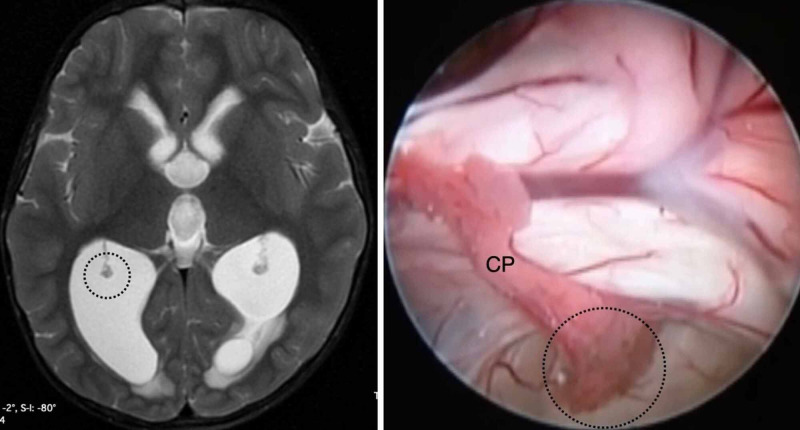
The type I anatomic variation of the glomus of choroid plexus (GCP). Type I GCP is classified as mobile, and the elongation is minimal. On both sides, the GCP (indicated by the black dotted circle) does not reach the middle of distance between the fixation and the posterior limit of the occipital horn, as observed in an axial view on T2-weighted magnetic resonance imaging (left). In this endoscopic view of the right lateral ventricle, the GCP is mobile and not amenable to resection. CP, choroid plexus.

Type I is characterized by a mobile GCP without excessive elongation (Figure 1).

**Figure 2:**
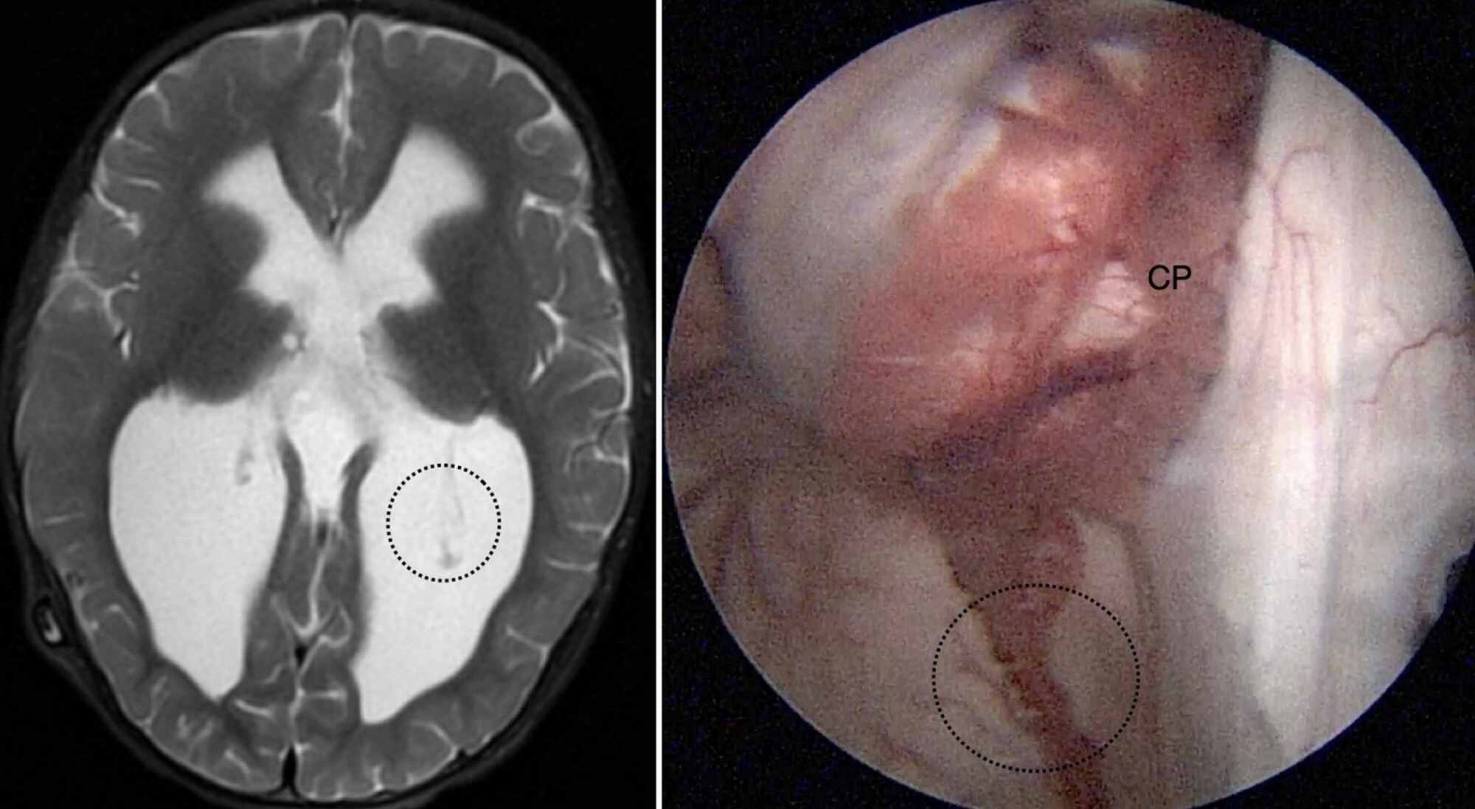
The type II anatomic variation of the glomus of choroid plexus (GCP). Type II GCP is classified as mobile, and the elongation is moderate. The glomus of left lateral ventricle reached the middle of distance between the fixation and the posterior limit of the occipital horn, as observed in an axial view on T2-weighted magnetic resonance imaging (left). In this endoscopic view of the left lateral ventricle, the GCP (indicated by the black dotted circle) is mobile and suitable for resection by our technique. CP, choroid plexus.

**Figure 3:**
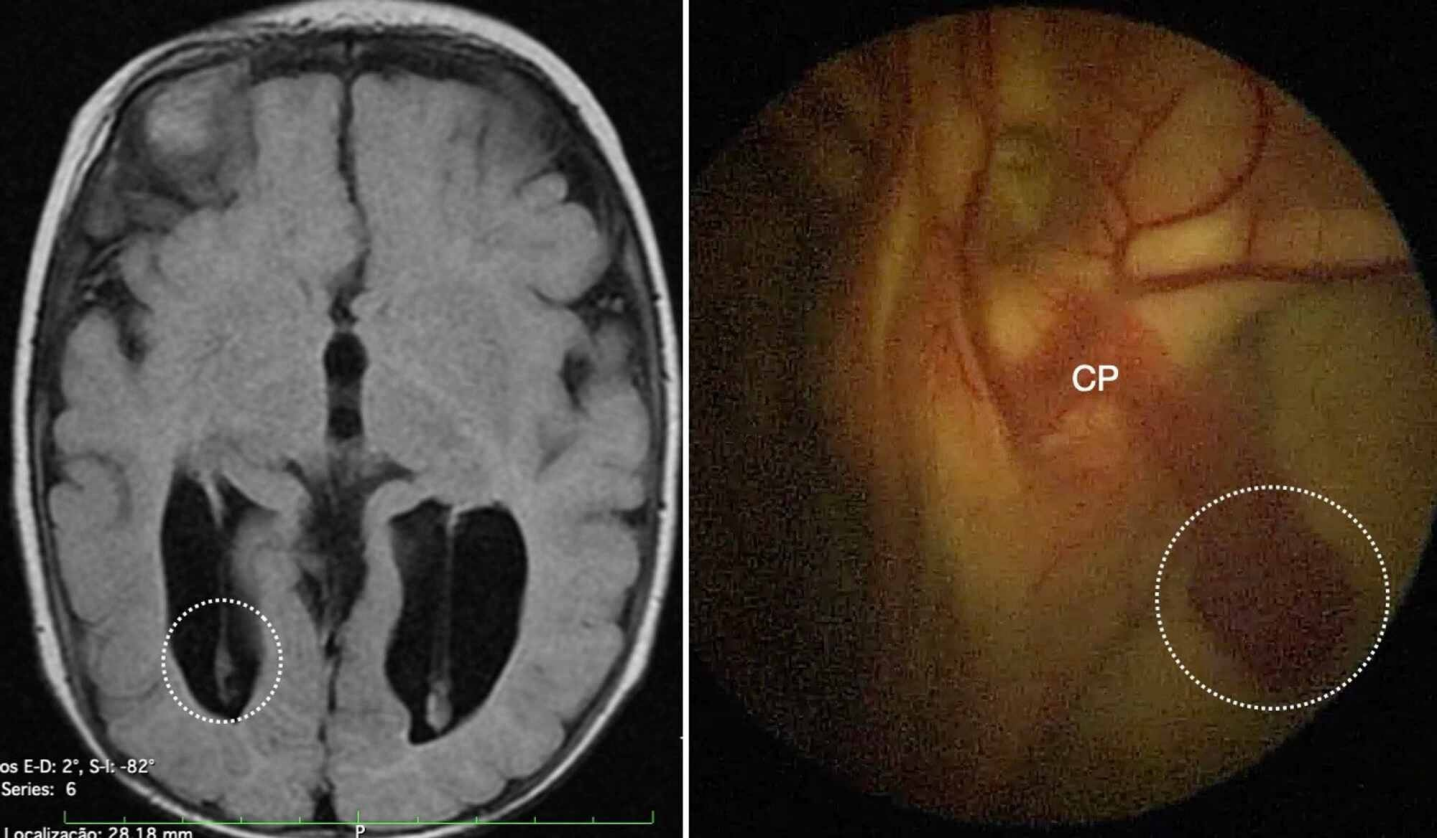
The type III anatomic variation of the glomus of choroid plexus (GCP). Type III GCP is classified as mobile, and the elongation is extreme. On both sides, the GCP (indicated by the white dotted circle) is closer to the posterior limit of the occipital horn, as observed in an axial view on T1-weighted magnetic resonance imaging (left). In this endoscopic view of the right lateral ventricle, the GCP is mobile and suitable for resection by our technique. CP, choroid plexus.

Both types II and III are characterized by an elongated GCP and are amenable to our technique (Figure 2, 3).

**Figure 4:**
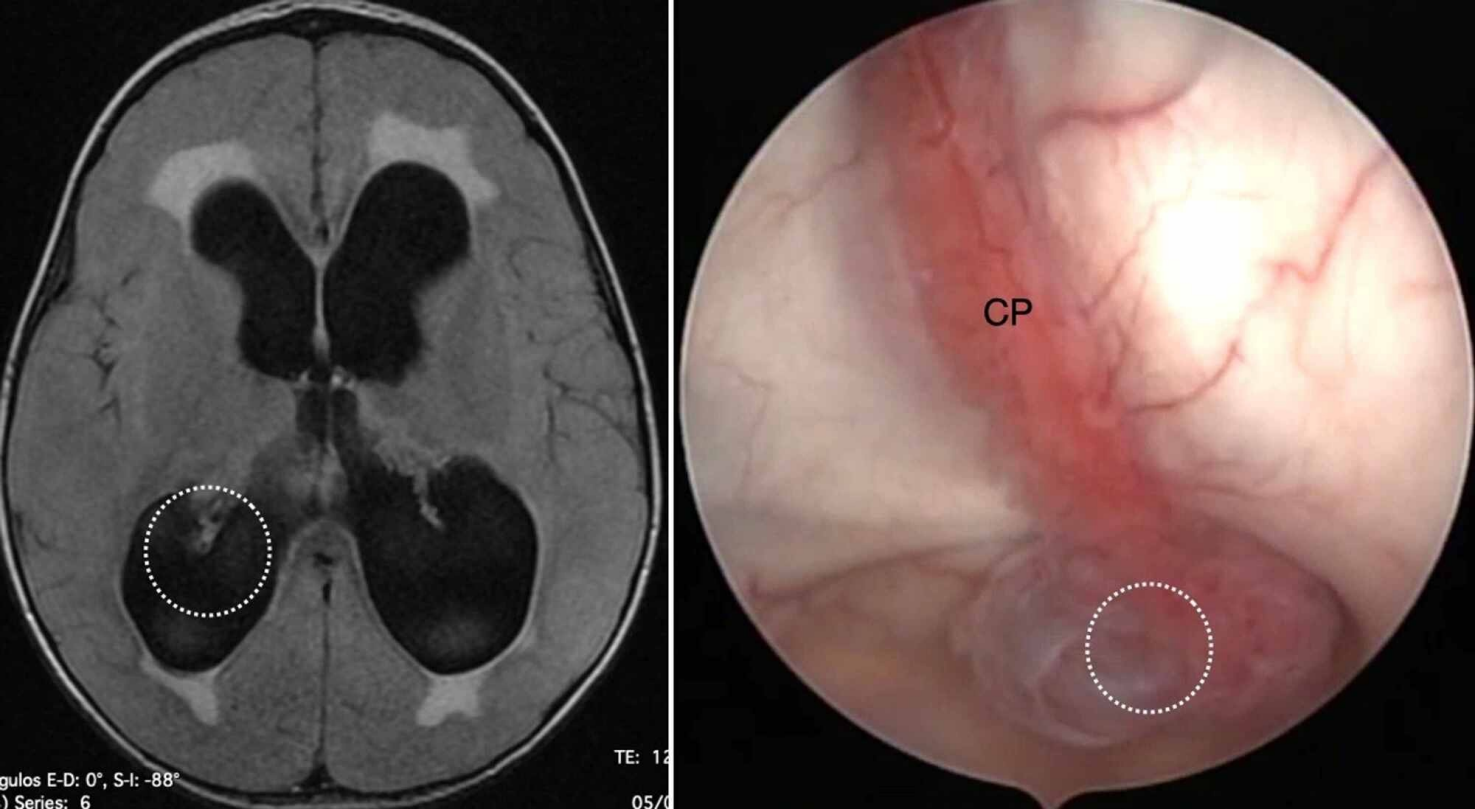
The type IV anatomic variation of the glomus of choroid plexus (GCP). Type IV GCP is classified as immobile and with no elongation. On both sides, the GCP (indicated by the white dotted circle) is close to it fixation near of pulvinar nuclei of the thalamus, as observed in an axial view on T1-weighted magnetic resonance imaging (left). In this endoscopic view of the left lateral ventricle, the GCP is a rounded tuft and unsuitable for resection by our technique. CP, choroid plexus.

**Figure 5:**
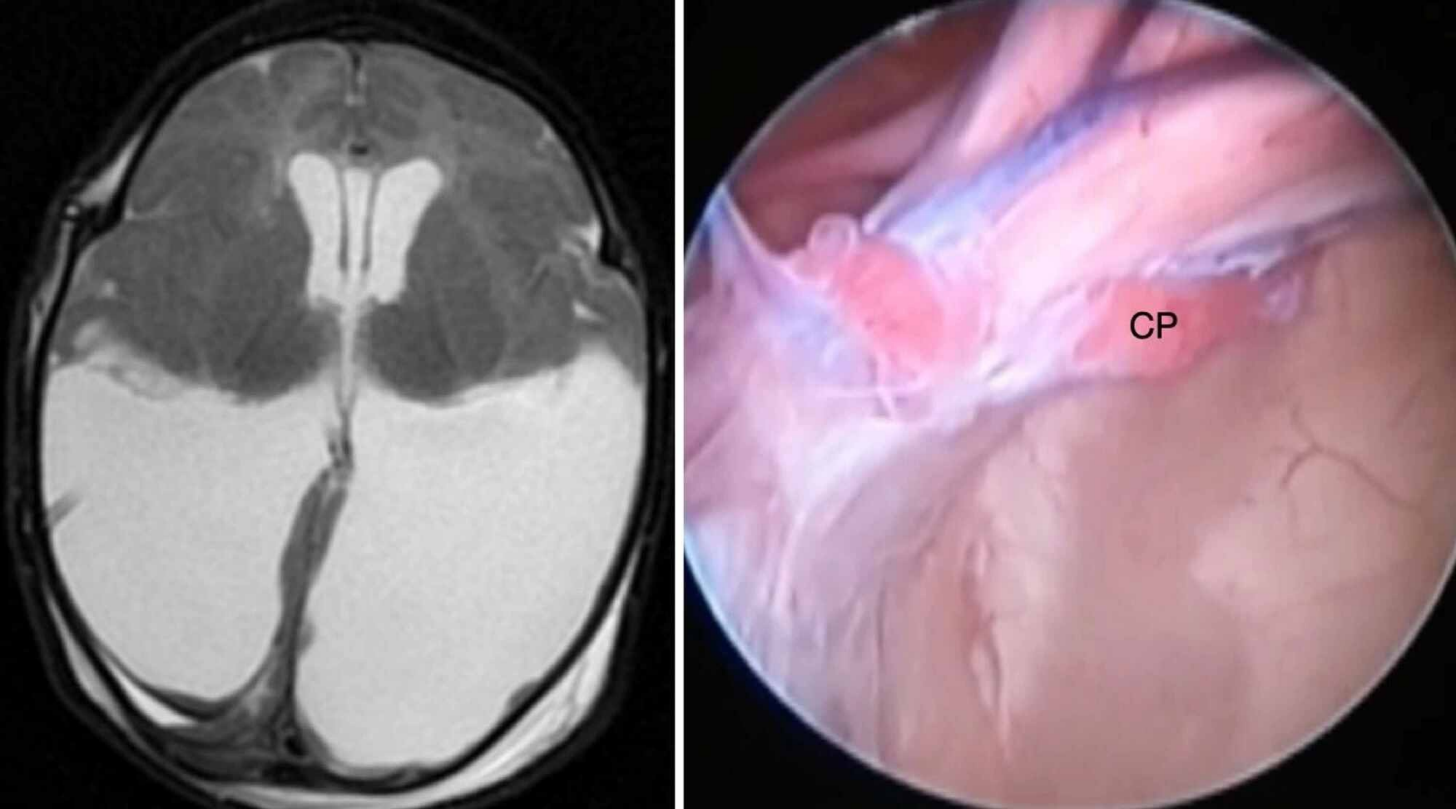
The type V anatomic variation of the glomus of choroid plexus (GCP). Type V GCP is classified as the absence of GCP. On both sides, the GCP is not visible in an axial view on T2-weighted magnetic resonance imaging (left). In this endoscopic view of the right lateral ventricle, the choroid plexus (CP) has the same appearance throughout the lateral ventricle with no glomus formation.

Type IV is characterized by a fixed GCP, and type V does not demonstrate any glomus formation (Figure 4, 5).

Surgical planning

**Figure 6:**
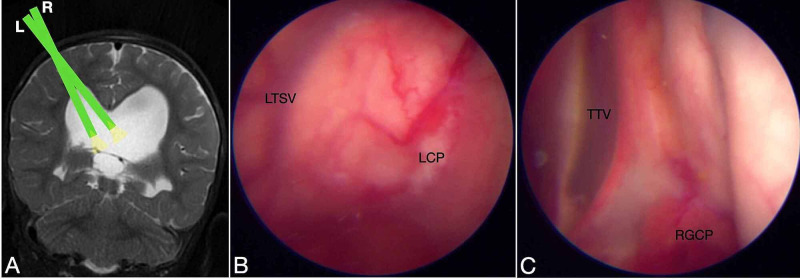
The bilateral ventricular view by an unique entry point The coronal view of a T2-Weighted magnetic resonance imaging (MRI) showed the lateral angulations of endoscope assessing the right (R) and left (L) lateral ventricles (A). In the same entry point, the endoscopic view enable to assess the left ventricle (B) and right ventricle (C). LTSV Left thalamostriate vein; LCP Left choroid plexus; TTV Tectum of third ventricle; RGCP Right Glomus of choroid plexus.

After assessing the anatomic features on MRI, we planned the surgery with a unique entry point for each patient (Figure 6).

**Figure 7:**
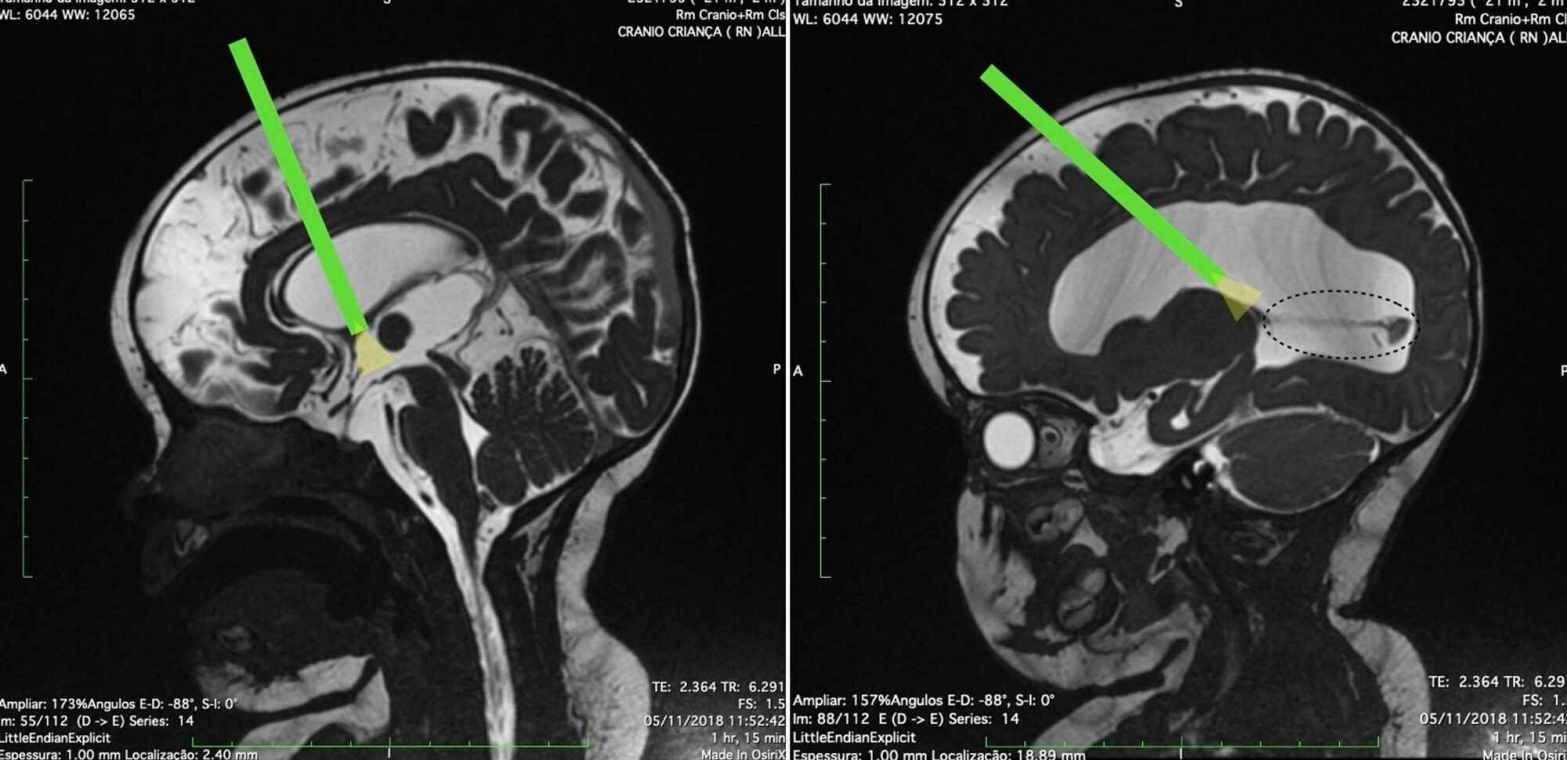
Planning of endoscopic third ventriculostomy (ETV) and choroid plexus cauterization with selective resection of the glomus in a patient with a type III variant. The sagittal view on T2-weighted magnetic resonance imaging (MRI) depicted the estimated angle of the endoscope during ETV (left). Through the same entry point, MRI allowed us to estimate the angle needed to reach the fixation of the glomus on the pulvinar nuclei of the thalamus (right). The green rectangle represents the endoscope, and the yellow triangle represents the illumination.

The first goal was to perform ETV, followed by CPC, and selective resection was to be performed at the end of procedure. At this time, the sagittal view on MRI helped us estimate the angle of the endoscope, which was to pass through the foramen of Monro first and would be angled posteriorly after ETV (Figure 7). 

Approach

Each patient was placed under general anesthesia and in a supine decubitus position with the head stabilized in a horseshoe headrest. The single entry point was on the right side, anterior to the coronal suture, and the skin was marked with methylene blue in a semicircle for the incision, with the concavity in the anterior direction. If a patient had a large and open anterior fontanel, we created an osteoplastic craniotomy in accordance with Costa Val et al.’s technique [5].

Endoscopic navigation

**Figure 8:**
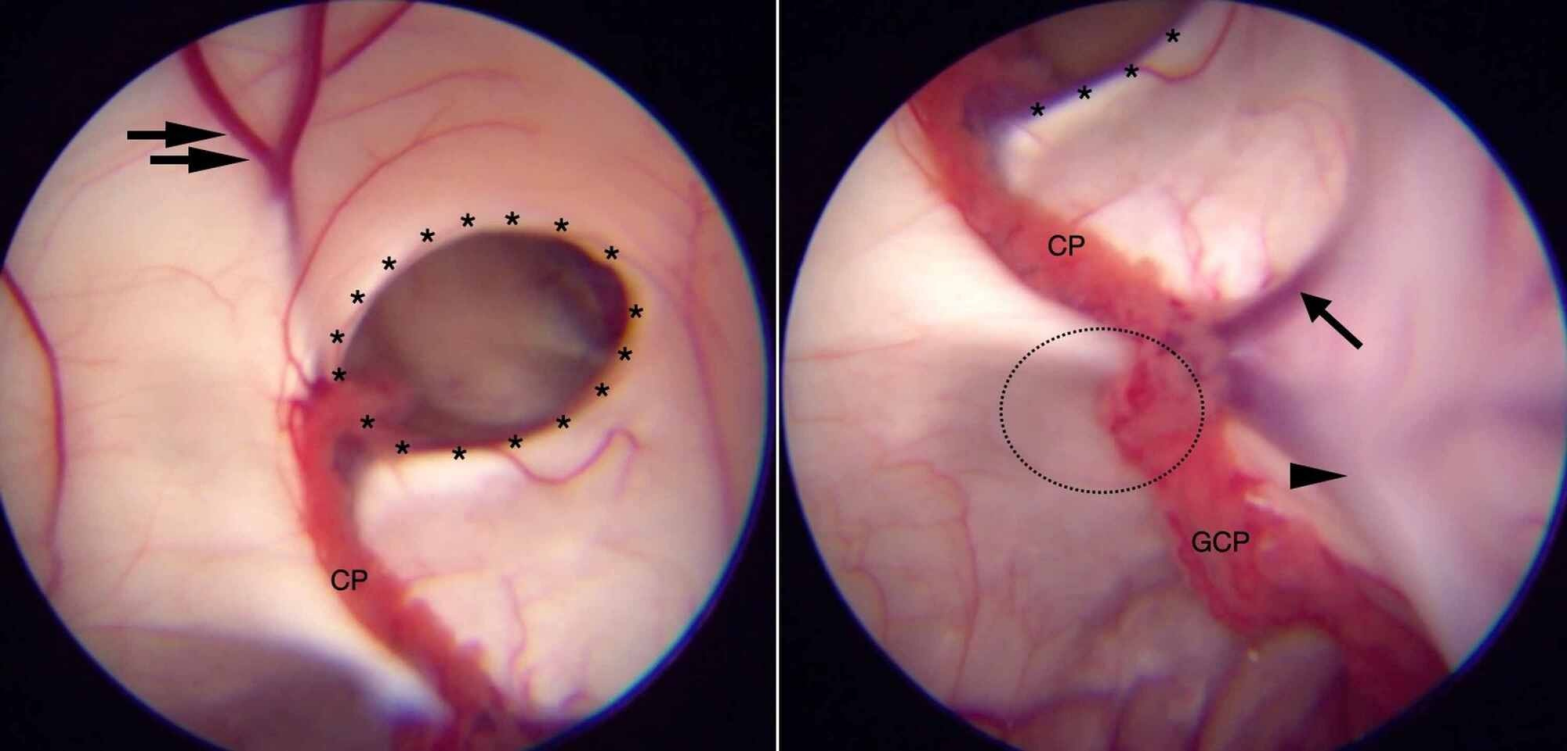
The anatomic landmarks of the lateral ventricle. This endoscopic view displays the main anatomic structures in the right lateral ventricle: the anterior septal vein (double black arrows), the foramen of Monroe (asterisk), the choroid plexus (CP) of the body of the lateral ventricle, the glomus of choroid plexus (GCP), the thalamostriate vein (single black arrow), the atrial lateral vein (Single black arrowhead). The pulvinar nuclei of the thalamus are represented by the black dotted circle.

After the right lateral ventricle was successfully punctured by a catheter with a peel-away sheath, the rigid endoscope (Aesculap, Center Valley, PA, USA) with a 0° angle was introduced, and we identified the main landmarks of the lateral ventricle, such as the choroid plexus, thalamostriate vein, anterior septal vein, septum pellucidum, and foramen of Monro (Figure 8).

The endoscopic third ventriculostomy was performed, and we confirmed the opening of the Liliequist membrane by visualizing the basilar artery through an ostomy.

Afterwards, the endoscope was retracted to the lateral ventricle, and the choroid plexus was coagulated with the use of bipolar diathermy. We did not use monopolar diathermy because it increases the risk of skin burns in small children, according to previous studies [6,7]. The coagulation started on the anterior edge of the choroid plexus in the foramen of Monro and progressed in a posterior direction along the right side. Afterwards, we performed pellucidotomy to achieve coagulation on the contralateral side.

During the entire procedure, the ventricles were continually irrigated by saline water warmed to the patient’s temperature. For extirpation of the choroid plexus, we coagulated the vessels of the tela choroidea after cutting them with a microscissor through the endoscope. The water flow exited through the endoscope, causing the GCP to move and spin, which facilitated further coagulation and disconnection. After the resection of the GCP, a biopsy forceps was used to maintain the catheter with the peel-away sheath in the ventricular puncture. Then the choroid plexus and the temporal horn were coagulated (Video 1).

**Video 1:** Selective extirpation of the choroid plexus. The endoscopic procedure was indicated in a girl with myelomeningocele that was diagnosed and repaired in utero. The patient developed symptoms of hydrocephalus (increased head circumference and bulging fontanel) at the age of eight months, and the endoscopic approach was indicated. Preoperative magnetic resonance imaging (MRI) depicted colpocephaly with elongation of the glomus of the choroid plexus (GPC; type III). She also had ventricular dilation (Evans index = 0.50 and fronto-occipital horn ratio [FOHR] = 0.54; not presented in this video). After endoscopic third ventriculostomy and coagulation of the choroid plexus on the right ventricle and the body of left ventricle, extirpation of the left GCP and further coagulation of remaining choroid plexus of temporal horn were performed. MRI one year later showed the maintenance of colpocephaly with no regeneration of the choroid plexus. The symptoms resolved completely with no complications.

At the end of the procedure, the irrigation was stopped, and the endoscope was withdrawn. The dura was stitched closed with Prolene 4-0 sutures (Ethicon, Inc., Somerville, NJ, USA), and fibrine glue was applied to the suture line. The skin was stitched closed by separated mononylon 3-0 sutures.

Results

**Figure 9:**
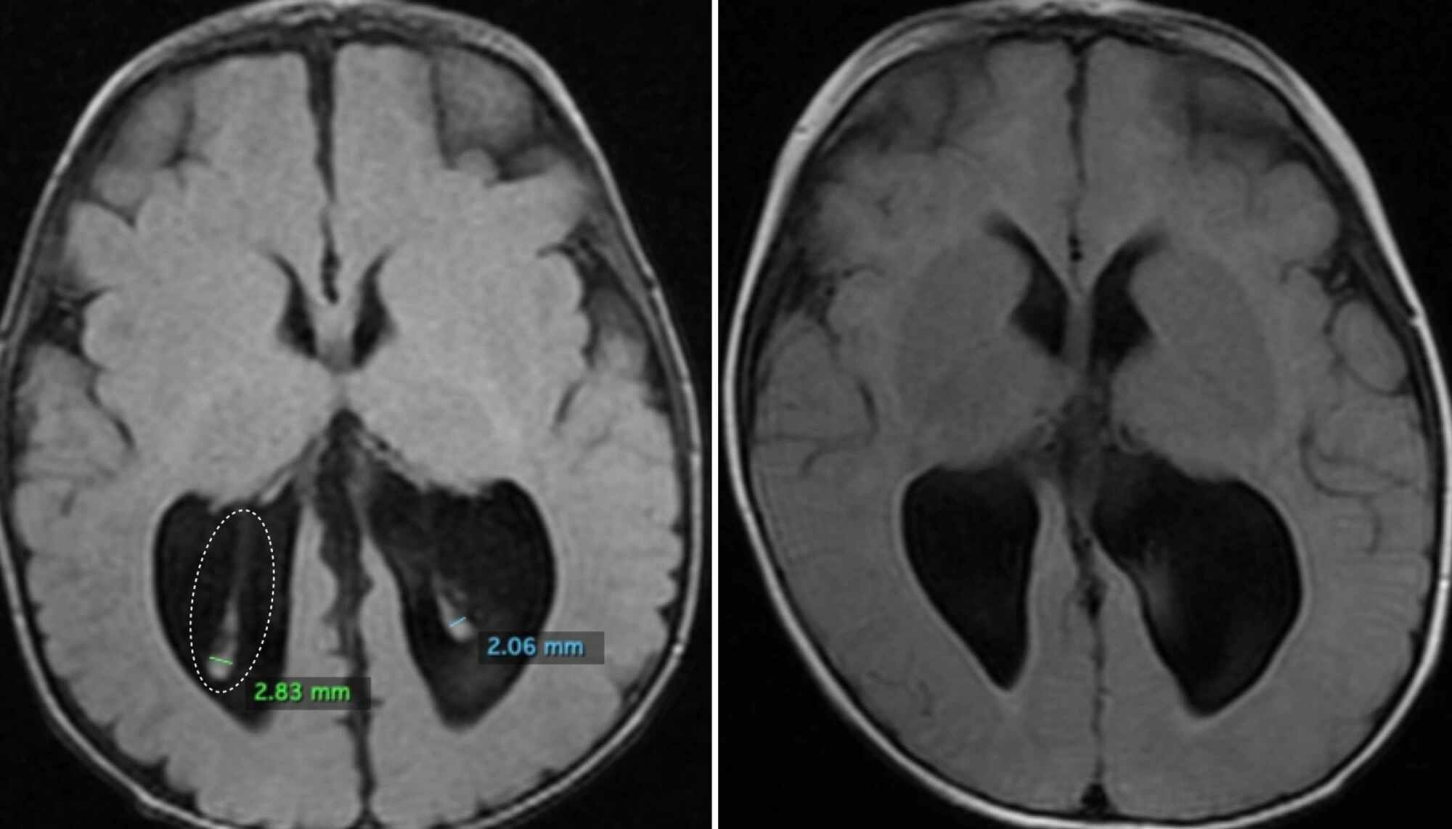
Outcome of selective choroid plexus resection. A: Axial T1-weighted magnetic resonance imaging before (left) and one year after endoscopic third ventriculostomy performed with choroid plexus cauterization and resection of the type III glomus of the choroid plexus (right). The patient was born with myelomeningocele, which was repaired in the first 24 hours after birth. Symptoms of hydrocephalus appeared when the patient was two months old, and the neuroendoscopic approach was indicated. The hydrocephalus symptoms resolved without a shunt, and imaging confirmed satisfactory control of cerebrospinal fluid.

We have performed this procedure in five patients (Table 2) during a mean follow-up period of two years, no complications and no intraoperative bleeding or deaths occurred. MRI in one of the patients that was obtained one year after this procedure is depicted in Figure 9 (see also Video 1).

**Table 2:** The features of five children underwent endoscopic resection of the glomus of choroid plexus combined to endoscopic third ventriculostomy (ETV) and choroid plexus cauterization (CPC) MMC Myelomeningocele; BF Bulging fontanelle; FW Follow-up; FTN Full Term Newborn

Patient	Gender	Age(Months)	Etiology	Indication	FW(Months)
1	M	1	Intraventricular hemorrhage (FTN)	Developmental delay	37
2	M	4	MMC (prenatal repair)	Parinaud syndrome	36
3	F	2	MMC (prenatal repair)	Macrocrania and BF	24
4	F	4	MMC (prenatal repair)	Developmental delay and BF	20
5	F	2	MMC (postnatal repair)	Macrocrania and BF	12

All patients demonstrated complete extirpation of the choroid plexus, even the part nearest the temporal horn.

## Discussion

Resection of the choroid plexus has been performed in an attempt to alleviate hydrocephalus, such as those with hydranencephaly [2,3,8-11]. This report is the first description of this modified technique and its effectiveness when used in combination with ETV and CPC. Since Dandy’s description of extirpation of the choroid plexus, researchers have reported variations in this procedure and the indications for it, but the majority have not used the endoscopic approach. Using a microscopic technique, Wellons et al. [12] performed plexectomy in children affected by hidranenephaly and reported a reduction in the need for reoperation and hospital admission. Lapras et al. [3] advocated plexectomy for patients with hydrocephalus and shunt infection. However, Milhorat et al. [10] highlighted the main disadvantage of plexectomy is the increased risk of ventricular collapse. We surmise that this risk is increased because this technique entails a longer duration of surgery and because of the loss of cerebrospinal fluid and subsequent development of pneumoventricle. Our technique of optimizing CPC and ETV by using the same bur hole does not extend the duration of surgery, and the constant irrigation prevents ventricular collapse and pneumoventricle, which occurred often during the early attempts at endoscopic extirpation.

Surgeons must recognize anatomic variations of the choroid plexus in order to select patients and enhance the safety of this technique. We have observed several discrepancies among classical anatomic descriptions, mainly in ex vivo samples from adults, and have observed many anatomic variations during ventricular endoscopy in children with many kinds of congenital and acquired diseases. According to the classical anatomic description, the choroid plexus of the lateral ventricles is fixed on the choroidal fissure in both sides by the taenia fimbriae and anchored to the fornix and the tela choroidea in the thalamus. The tela choroidea in the thalamus is more vascularized than the other structures. The choroid plexus extends from the foramen of Monro to the temporal horn in the inferior choroidal point. The GCP was described as a triangular tuft that occupies the atrium of the lateral ventricles and is fixed in the portion of the choroid fissure near the pulvinar nuclei in the thalamus [13,14]. In an endoscopic view, the anatomic appearance of the GCP is quite heterogeneous, as our classification proposed for this technique implies. For the procedure described in this report, this classification applied for the first time. Further studies are needed to determine whether this classification would have implications about the effectiveness of hydrocephalus treatment and its real incidence.

**Figure 10:**
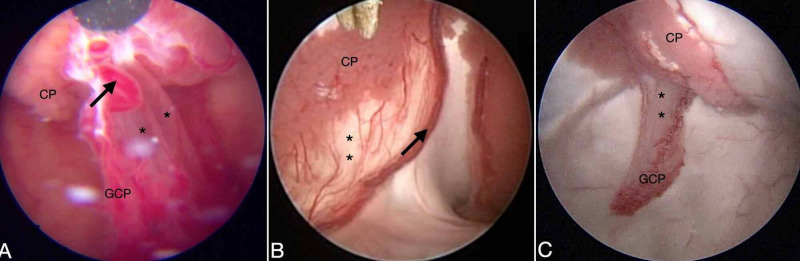
The choroid plexus fixation on the lateral ventricles. A large choroidal artery (black arrow) is observed in the glomus of the choroid plexus (GCP), and the tela choroidea (asterisk) is perceived as a translucid membrane (A). The patient was born with agenesis of the septum pellucidum, but the choroid plexus is well separated from the choroidal artery by the tela choroidea (B). The GCP appeared as an elongation here (C).

Another difference from the classical description of choroid plexus anatomy was the degree of fixation of the choroid plexus in the choroidal fissure. The endoscopic view enables us to identify several patterns of elongation of tela choroidea as well as different shapes of vessels and the density of the choroid plexus (Figure 10).

The main advantage of extirpation of the GCP in addition to CPC is the improvement in surgical efficiency because this portion of the choroid plexus is more mobile than other segments, especially in patients with myelomeningocele and colpocephaly [15]. Neuroendoscopic procedures involve only one portal, and the tip of a monopolar or bipolar diathermy device frequently adheres to the glomus, thereby increasing the risk of bleeding and lengthening the procedure. Hemorrhage should be avoided during this procedure, and we recommend using the bipolar device in the tela choroidae of the glomus and cutting the glomus progressively with the maximum approximation of the endoscope. We also recommend selecting patients in whom the glomus is adequately elongated with clear visualization of vessels of the tela choroidae to facilitate coagulation and division, such as those designated types II and III in our classification.

The variations in ventricular anatomy observed in patients with myelomeningocele have been reported in some studies [16,17]. The most common findings are large interthalamic adherences, the presence of interhypothalamic adherences, the absence of the septum pellucidum, and anomalies of the choroid plexus, which vary from its absence to being large. We advocate the use of our adapted technique during CPC in order to shorten operating time and avoid bleeding that could result from difficulties related both to endoscopic maneuvers in the region of the lateral ventricular atrium and to the peduncular appearance of the choroid plexus.

Our technique evolved from the disconnection of the GCP with no bleeding and as an adjuvant technique during CPC in patients with myelomeningocele. Another advantage of glomus extirpation is that CPC can be extended in the temporal horn, even with a rigid endoscopic. Warf et al. [18,19] demonstrated an increase in the success rate (from 43% to 76%) when CPC was performed with ETV with the use of a flexible endoscope and when CPC was performed in the temporal horn. According to Fallah et al. [20], the extension of CPC could be classified as partial, unilateral, or bilateral when the atrium is reached in at least one side. CPC was considered subtotal when the atrium is reached bilaterally and complete when the temporal horn tip is reached bilaterally. However, Fallah et al. did not find a statistical difference between subtotal and partial effectiveness.

Our technique does have some limitations. It could be performed only with specific anatomic configurations of the glomus; otherwise, the safety of coagulation of the tela choroidae and resection of glomus with no bleeding could not be guaranteed. The presence of colpocephaly and large ventricles facilitates this procedure. Instruments such as scissors and biopsy forceps must be used, and expertise in ventricular neuroendoscopy is required.

## Conclusions

Intraventricular neuroendoscopy have evolved considerably with the development of surgical techniques and the endoscopic armamentarium. The endoscopic extirpation of the GCP is an example of how neuroendoscopic techniques can enhance classical techniques such CPC and thereby optimize the treatment of hydrocephalus in selected patients.
